# Zinc-alpha 2 glycoprotein a diagnostic Biomarker for early stage oral Squamous Cell Carcinoma

**DOI:** 10.12669/pjms.39.2.6488

**Published:** 2023

**Authors:** Mehwish Feroz Ali, Mervyn Hosein, Saima Butt, Rehan Siddiqui

**Affiliations:** 1 Dr. Mehwish Feroz Ali, BDS, MPhil-Oral Pathology. Assistant Professor, Department of Oral Pathology, Ziauddin University, Karachi, Pakistan; 2 Dr. Mervyn Hosein (BDS, FCPS) Principal Ziauddin College of Dentistry, Ziauddin University, Karachi, Pakistan; 3 Dr. Saima Akram Butt (BDS, MDS, Head of Oral Pathology Department, Ziauddin College of Dentistry, Karachi, Pakistan; 4 Dr. Rehan Ahmed Siddiqui (PhD), Assistant Professor, Research Department, Ziauddin University, Karachi, Pakistan

**Keywords:** Zinc alpha-2 glycoprotein, Mouth neoplasm, Adipokines, Neoplasm staging, Immunohistochemistry

## Abstract

**Objectives:**

In this study, we investigated the expression of zinc alpha-2 glycoprotein in oral squamous cell carcinoma tissue samples. Additionally, ascertained its association to the oral cancer stage and subscale parameters (TNM).

**Methods:**

This observational study was conducted at Ziauddin University from January to December 2020. Using the Open-Epi software, the sample size of 120 oral squamous cell carcinomas was calculated at 95% confidence interval and a 5% margin of error. Ethical approval was taken from the Institutional Ethical Review Committee. Histologically diagnosed cases of oral squamous cell carcinoma were obtained from the Histopathology Department of Ziauddin University, Karachi. Study data was analyzed through SPSS version-20 and p-value ≤0.05 considered as significant. One-way ANOVA and Multiple linear regression were applied for analysis of data.

**Result:**

In the study, none of the oral squamous cell carcinoma tissue samples from the later stages were stained for ZAG. However 71% (35/49) of the early stage OSCC samples showed positive IHC results for ZAG expression in the cytoplasm. One-way ANOVA indicates that high ZAG expression was significantly associated with smaller tumor size (p<0.001), lymph node involvement (p=0.002), early stages of OSCC (p<0.001) and less differentiated tumor (p=0.001). The site of the tumor was also significantly associated with ZAG staining (p<0.001).

**Conclusion:**

Zinc alpha-2 glycoprotein expressed in the early stages of oral cancer development so that effective treatment modalities can be planned as per the patient’s status. This may also assist a clinician to achieve tumor-free surgical margins and monitor the post treatment outcomes.

## INTRODUCTION

Oral squamous cell carcinoma (OSCC) is the most common type of head and neck cancer, accounting for 90% of all oral cancers.^1^ According to the literature, the global occurrence of oral cancer is 2-4 percent, 10.9 percent in Pakistan, and 40 percent in India.^2^,^3^ This could be attributed to increased consumption of both smoked and smokeless tobacco in South-East Asia.^4^ Despite advanced treatment options, treatment outcomes are unsatisfactory due to tumor invasion, metastasis and recurrence.^5^ There is a scope to identify a potential biomarker that have tumor suppressor activity, inhibits degree of differentiation and can improve patients’ prognosis. The biomarker can assist a clinician to plan treatment, use it as adjuvant and monitor the post-treatment outcomes.^6^

In this study, we looked at the expression of Zinc alpha 2-glycoprotein (ZAG) in oral squamous cell carcinoma tissue samples and how it correlated with clinical and histological parameters. ZAG is a novel adipokine which is secreted by adipose tissue, liver, epithelial ductal cells and the tumor itself.^7^ ZAG induces lipid catabolic activity directly in adipocytes by a cyclic AMP-mediated process and this is initiated through binding to a *β3-adrenoceptor*.^8^ This is due to its high structural and functional resemblance to lipid mobilizing factor, with the exception of a minor difference in post-translational modification.^7^ Vidoto et al. have found increased expression of ZAG in head and neck cancer, suggesting that the high levels of ZAG inhibit tumor cells proliferation via immune activity against tumor antigen.^9^ According to Hasan et al, ZAG inhibits enzyme-mediated tumor invasion and proliferation due to its high structural and functional similarity to class I MHC molecules and its complex with enzymes (macroglobulin/hydrolases).^10^

According to studies, high expression of ZAG is associated with elimination of mutated RNAs and their by-products, as well as the down-regulation of cyclin-dependent kinase 2 (rate limiting enzymes) in the cell cycle.^11^ The enzyme is required to regulate the G2-M phase of the cell cycle, and by doing so, it may contribute to the inhibition of tumor cell growth. ZAG overexpression promotes epithelial-mesenchymal transition (EMT), tumor invasion, and apoptosis via the TGF1-ERK2 signaling pathway.^12^ This could be accomplished by down-regulating epithelial markers (E-cadherin) and increasing mesenchymal markers (N-Cadherin).

According to the literature, ZAG has been found in a variety of tumors including oropharyngeal, esophageal, gastric, breast, prostate, pancreas, and liver tumours.^9^-^11^ The up-regulation of ZAG in various cancers was observed in early tumor stages, which was associated with a longer disease free survival and overall survival rate.^11^-^13^ There is a discrepancy in the literature about ZAG expression in tumors and its role as an early diagnostic marker. There have been very few studies on its expression in oral squamous cell carcinoma.^13^-^15^ In this study, we assessed ZAG expression in the tissue samples of oral squamous cell carcinoma cases. And also determine its correlation with staging and subscale parameters (TNM) of oral cancer.

## METHODS

This observational study was conducted at Ziauddin University from January to December 2020. The sample size of 120 cases of oral squamous cell carcinoma was calculated using Open-Epi software with a 95% confidence interval and a 5% margin of error. The Ziauddin University Ethical Review Committee provided ethical approval (Reference code: 1531010MFOM). The sampling method used was consecutive. The Histopathology Department of Ziauddin University, North Campus, Karachi, provided histologically diagnosed cases of oral squamous carcinoma. By obtaining informed consent from each department, all samples were included in the study.

Cases of OSCC that had been surgically removed and histologically determined match the inclusion criteria. Patients aged ≥18 were included in the study. Recurrent OSCC, malignancy other than OSCC, improperly stained histological slides, and a paucity of tissue in paraffin blocks are all exclusion criteria. The paraffin-embedded tissue blocks were sectioned into 3-5µm wide slices. The tissue sections were placed on the glass slide. Two glass slides were prepared, one for IHC and another slide for hematoxylin & eosin staining. The expression of zinc α-2 glycoprotein was investigated by Immunohistochemical staining using biotinylated antibodies against ZAG antigen on the cancer tissue sample.

The cases were graded by using WHO/Broder classification and staging was evaluated by using the AJCC (TNM classification). The AZGP1 polyclonal antibody kit was purchased from the Thermo-scientific company. The standard protocol of IHC was applied. The histopathologist found 20 areas which were distributed among the marked hot spots with high brown staining. Each area 80% covered by tumor cells without any artifact. The IHC slides were interpreted as ZAG positive cells scores as 0; <5%, 1; 5%-25%, 2; 25%-50%, 3; 50%-75%, and 4; >75%. The ZAG positive staining intensity is scored as 0(-) Negative, 1(+) Mild, 2(++) Moderate, and 3(+++) Strong. Slides were examined under Ts2R-FL inverted research microscope (Nikon). Images were captured using NIS element D software. Images were processed using Photoshop.

The following variables were recorded: gender, age, site of the tumor, size and thickness of the tumor, nodal involvement, distant metastasis, clinical stages, histological grades, lymphovascular invasion, perineural invasion, and extracapsular spread. Statistical analysis was performed by SPSS version 20.0 (SPSS Inc., Chicago, USA). The frequency and percentage were calculated for the categorical data. Mean and SD for the quantitative data. One-way ANOVA and Multi-regression analysis were used to find correlation of ZAG staining intensity with clinical and histological features. P-value ≤0.05 was considered statistically significant.

## RESULTS

In the study, there were 100 (83.3%) males and 20 (16.7%) females. The maximum OSCC cases fell within the third (36; 30%) and fourth (33; 27.5%) decades of life. The most common site was buccal mucosa (80; 66.7%) followed by the lateral border of the tongue (21; 17.5%) and alveolar mucosa (9; 7.5%). The mean age was 47.2±11.1 years, the mean size of the tumor was 3.7 ± 1.9 mm, and the mean thickness was 1.5 ± 1.3 mm. In the reported cases of OSCC, there were 90 (75%) cases of moderately differentiated OSCC, 18 (15%) well differentiated and 12(10%) of poorly differentiated. Majority of patients presented at the late stages of OSCC around (71) 59%.

None of the advanced stage OSCC tissue samples observed to be positive for ZAG staining on IHC but 71% (35/49) early stage OSCC samples showed positive staining of ZAG. The positive ZAG staining in the tissue samples of OSCC through Immunohistochemistry (IHC) is represented in Fig-I. The ZAG protein was expressed in the cytoplasm of the tumor cells. The image A shows ZAG staining of mild intensity (1+) in the cytoplasm of the oral cancer cells around 25-50% tumor cells that were positively stained. The image-C shows staining of moderate intensity (2++) about 50-75% oral cancer cells that were positively stained. The image-E shows staining of strong intensity (3+++) >75% oral cancer cells that were positively stained. The images B, D & F were the hematoxylin and eosin counterparts of the IHC slides.

**Fig.1:**
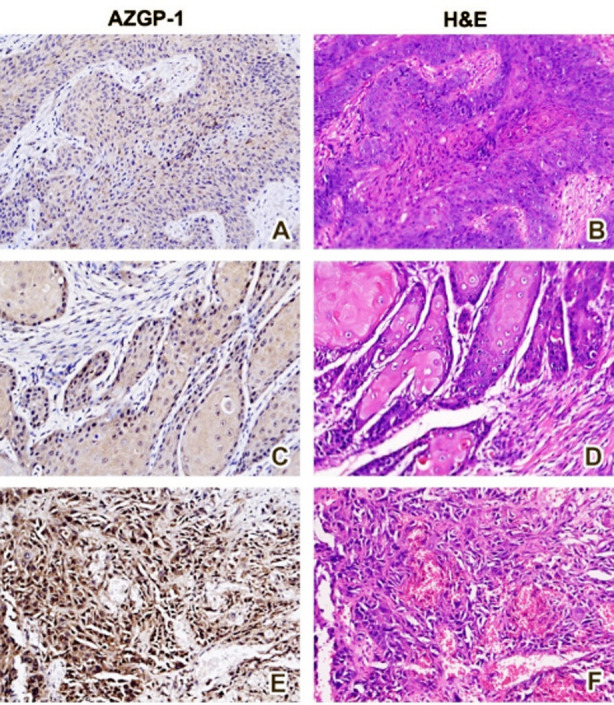
Images A, C & E represents the Immunohistochemical staining (IHC) of ZAG in oral squamous cell carcinoma tissue samples with hematoxylin and eosin counterstain (images B, D & F). The image A shows ZAG staining of mild intensity (1+) in the cytoplasm of the oral cancer cells around 25-50% tumor cells that were positively stained for ZAG. The image C shows staining of moderate intensity (2++) about 50-75% oral cancer cells that were positively stained for ZAG. The image E shows staining of strong intensity (3+++) >75% oral cancer cells that were positively stained for ZAG.

The association of staining intensity with clinicopathologic parameters of OSCC patients by using One-way ANOVA is represented in Table-I. The statistics indicate that high ZAG expression was significantly associated with smaller tumor size (p<0.001), lymph node involvement (p=0.002), early stages of OSCC (p<0.001) and less differentiated tumor (p=0.001). The site of the tumor were also significantly associated with ZAG staining (p<0.001). Moreover, ZAG didn’t show any association with gender (p=0.097), and age (p=0.889). To confirm the correlation of ZAG staining with staging and grading of OSCC, multiple linear regression method was applied which showed statistically significant results (p<0.001, p=0.001) respectively.

**Table-I:** Showing correlation between intensity of ZAG staining with staging of OSCC.

Clinical Staging	Staining Intensity				Total

	Negative	Weak	Moderate	Strong	
Stage I	2	6	13	1	22
Stage II	15	5	6	1	27
Stage III	23	0	0	0	23
Stage IV	45	3	0	0	48
Total	85	14	19	2	120
p-value	<0.001*				

* Spearman correlation showed a statically significant result.

**Table-II:** Represents the association of ZAG staining with various clinic-pathologic parameters of OSCC patients by using One-way ANOVA.

Variables		Sum of Squares	df	Mean Square	F	Sig.
Clinical Staging	Between Groups	75.939	3	25.313	35.526	.000*
	Within Groups	82.652	116	.713		
	Total	158.592	119			
Grading	Between Groups	4.069	3	1.356	6.139	.001*
	Within Groups	25.631	116	.221		
	Total	29.700	119			
Age	Between Groups	.882	3	.294	.210	.889
	Within Groups	162.318	116	1.399		
	Total	163.200	119			
Site	Between Groups	77.047	3	25.682	7.244	.000*
	Within Groups	411.278	116	3.546		
	Total	488.325	119			
Gender	Between Groups	.881	3	.294	2.157	.097
	Within Groups	15.786	116	.136		
	Total	16.667	119			
Tumor size (T)	Between Groups	42.350	3	14.117	14.939	.000*
	Within Groups	109.617	116	.945		
	Total	151.967	119			
Nodal Involvement (N)	Between Groups	49.702	3	16.567	5.151	.002*
	Within Groups	373.090	116	3.216		
	Total	422.792	119			
Metastasis (M)	Between Groups	.454	3	.151	1.192	.316
	Within Groups	14.713	116	.127		
	Total	15.167	119			

* P-value ≤0.05 was considered statistically significant.

## DISCUSSION

In the study, the zinc alpha-2 glycoprotein was positively expressed in the early stages of OSCC tissue samples. Similarly, studies have found that high levels of zinc alpha-2 glycoprotein expression are associated with a lower degree of differentiation in many cancers.^9^-^15^ Though the precise mechanism is unknown, ZAG expression has been regulated by acetylation of histone which controls genes through chromatin conformation.^16^ Another research found that histone deacetylation leads ZAG expression to be decreased in pancreatic cancer.^16^ Zinc alpha-2 glycoprotein is a clinically significant protein that plays a role in tumor formation and proliferation.^9^-^16^ It was first identified as a lipid mobilizing factor, subsequently as a tumor marker that increases in disease-specific cachexia in humans.^17^,^18^ The increased level of ZAG mRNA and protein in cancer cachexia occurred by peroxisome-activated receptor gamma (PPARγ) and uncoupling protein (UCP-2).^7^ The down-regulation of ZAG usually associated with aggressive patterns of tumor proliferation, poor prognosis and worse treatment outcomes.

In the study, a multiple linear regression analysis was used to assess the association between ZAG and many clinical and pathologic parameters. The majority of ZAG positive stained samples were from men, owing to the fact that men in our demographic consume the most smokeless tobacco.^2^ The buccal mucosa was shown to be the most common positive site for ZAG staining in the study. The explanation for this is the use of powdered tobacco in one or more forms. It is typical practice in our population to place powered tobacco in the buccal sulcus, which releases its content locally.^5^ This results in continual mucosal irritation, chronic inflammation, and premalignant and cancerous alterations.^3^

In the study, we investigated how ZAG correlated with histological components such histological grades, lymphovascular invasion, perineural invasion, and extracapsular spread to predict patient prognosis. ZAG was not expressed in any of the OSCC cases that had lymphovascular invasion, perineural invasion, or extracapsular spread. Poropatich et al also found an up-regulation of ZAG in the low grade oropharyngeal squamous cell carcinoma with significantly longer recurrence free survival.^17^ According to Vidotto et al, increased ZAG expression induces an immune response against tumor antigens and mucosal breakdown by proteolytic enzymes in Head and Neck Squamous Cell Carcinoma.^9^ This could imply that ZAG inhibits tumor proliferation and differentiation. This could also suggest that reduction of ZAG expression is linked to metastasis, poor prognosis, local recurrence, and unsuccessful treatment outcomes.

On multivariate analysis, greater ZAG expression was associated with smaller tumor size T1 and T2, as well as no lymph node metastases in the study. According to a study conducted by Haung et al, ZAG protein downregulation was correlated with larger tumor size but not with nodal involvement or distant metastasis.^19^ This indicates that the more oral tissues involved in the malignant process, the lower the expression of ZAG. According to Xu et al study, low ZAG levels in hepatocellular carcinoma cell lines enhance epithelial to mesenchymal transition (EMT) via the TGF1-ERK2 signaling pathway.^20^ This could occur by down-regulating epithelial markers (E-cadherin) and up-regulating mesenchymal markers (N-Cadherin).^20^ This may be linked to the fact that less differentiated tumors in the study did not express positive ZAG staining.

Tang and colleagues have reported down-regulation of ZAG in the aggressive stages of esophageal squamous cell carcinoma, which could indicate increased rate of tumor progression.^16^ ZAG overexpression activates the rapamycin signaling pathway and inhibits the cyclin dependent 2 kinase enzyme, which controls the cell cycle’s G2-M transition.^22^ It inhibits tumor cell growth, proliferation, and migration in this way.^22^,^23^ In contrast, Dengbo et al. study reported high levels of ZAG in the advanced stages of colorectal carcinoma and associated with hepatic metastasis, shorter disease free survival and overall survival.^24^,^25^ All of the aforementioned studies suggested that ZAG may play a role in the initiation and progression of carcinomas.^16^-^25^ In future, ZAG could be used as a reliable biomarker to maintain epithelial phenotype, predict prognosis, and post-treatment outcomes.

To the best of our knowledge, we have investigated ZAG expression for the first time in oral squamous cell carcinoma tissue samples. The findings demonstrated a significant expression of ZAG in the early stages of OSCC, suggesting that it may be a useful marker for predicting the prognosis and effectiveness of treatment in such patients. It could serve as a therapeutic agent or assist a clinician plan a course of treatment according to the ZAG expression. However, further thorough investigations are warranted to find the precise role of the ZAG in oral squamous cell carcinoma.

### Limitations:

It includes that only one method was used to assess the ZAG expression in the tissue samples due to financial restraints. Because of the same reason we were also unable to perform protein validation in our study.

## CONCLUSION

The zinc alpha-2 glycoprotein positive stained samples in this study belonged to the early stages of oral squamous cell carcinoma. Positively stained samples are less differentiated, showing that ZAG expression decreased with increased tumor cell proliferation and differentiation. The zinc alpha-2 glycoprotein may be used to diagnose OSCC in its early stages and to plan treatment modalities based on the needs of the patient. This may also aid a surgeon in achieving tumor-free surgical margins. According to the study’s findings, ZAG could be used as a potential biomarker for prognosis and post-therapy outcomes in oral cancer.

### Authors’ contributions:

**MF:** Conceived and designed the study, collected the data, performed the analysis, and writing of the manuscript.

**MH:** Conceived and designed the study, clinical integrity of the study, interpretation of data, and proof-reading.

**SB & RS:** Conceived and designed the study, interpretation of data, and proof-reading.

All authors are responsible and accountable for the accuracy and integrity of the work.
